# Parthenolide suppresses non-small cell lung cancer GLC-82 cells growth via B-Raf/MAPK/Erk pathway

**DOI:** 10.18632/oncotarget.15584

**Published:** 2017-02-21

**Authors:** Minting Lin, Hong Bi, Yanyan Yan, Wenjing Huang, Guiping Zhang, Genshui Zhang, Sili Tang, Yun Liu, Lingling Zhang, Jinxiang Ma, Jianye Zhang

**Affiliations:** ^1^ School of Pharmaceutical Sciences and The Fifth Affiliated Hospital, Guangzhou Medical University, Guangzhou 511436, People's Republic of China; ^2^ Department of Pathology, Shanxi Provincial People's Hospital, Taiyuan 030012, People's Republic of China; ^3^ Institute of Respiratory and Occupational Diseases, Collaborative Innovation Center for Cancer, Medical College, Shanxi Datong University, Datong 037009, People's Republic of China; ^4^ Pharmaceutical Department, The Fifth Affiliated Hospital of Guangzhou Medical University, Guangzhou 510700, People's Republic of China; ^5^ School of Public Health, Guangzhou Medical University, Guangzhou 511436, People's Republic of China

**Keywords:** non-small cell lung cancer, parthenolide, B-Raf, MAPK/Erk pathway, c-Myc

## Abstract

Non-small cell lung cancer (NSCLC), one type of lung cancer, owns high rates of morbidity and mortality. B-Raf is one of the promising oncogenic drivers of NSCLC. Parthenolide, a natural product, is mainly extracted from the herbal plant *Tanacetum parthenium*. The effect of parthenolide on NSCLC cells and its potential as B-Raf inhibitor were studied in this study. It's shown that parthenolide exhibited the strong cytotoxicity against NSCLC cells with IC50 ranging from 6.07 ± 0.45 to 15.38 ± 1.13 μM. Parthenolide was also able to induce apoptosis, suppress proliferation and invasion in NSCLC cells. In terms of the involved mechanism, parthenolide suppressed GLC-82 cell response via targeting on B-Raf and inhibiting MAPK/Erk pathway signaling. The effect of parthenolide on B-Raf and MAPK/Erk pathway was further confirmed by RNA interference of B-Raf. Decreased expression of c-Myc in protein and mRNA level was also discovered, which is considered as the further downstream of the MAPK/Erk pathway. In addition, STAT3 activity inhibition by parthenolide contributed to its effect on GLC-82 cells, which is independent of PI3K pathway signaling and GSK3. All above provide an insight to understand the action of parthenolide as a potential B-Raf inhibitor in treatment of NSCLC.

## INTRODUCTION

Based on WHO estimates, cancer now causes more death than all coronary heart disease or all stroke [1]. Lung cancer, among different kinds of cancers, remains the most common cancer in the world, both in term of new cases (1.8 million cases, 12.9% of total) and death (1.6 million deaths, 19.4%) because of the high case fatality [2, 3]. It is usually divided into two categories, non-small cell lung cancer (NSCLC) and small cell lung cancer (SCLC), of which the former accounts for about 80%. Surgery, radiotherapy, chemotherapy, molecular targeted therapy and combined treatment of them are the common treatments for NSCLC. However, most of NSCLC patients were locally advanced and advanced at their first visit, with five years survival rates lower than 10% and 5% [4]. Drug therapy, including chemotherapy and molecular-targeted therapy, is the main treatment for this staging and plays an important role in it.

Thoracic oncology has witnessed an unprecedented outburst of knowledge regarding molecular biology of NSCLC during the last decade. A number of oncogenic drivers, such as *EGFR*, *ALK*, *KRAS*, *B-RAF*, have emerged as novel molecular targets with potential therapeutic implications [5, 6]. Molecular targeted therapy, due to its high efficiency and low toxicity, has received more and more attention [7].

Natural products from Chinese herbal medicine are important source of anti-cancer drug development. It's reported that nearly 67% of the anti-cancer drugs are natural products or natural products derivatives and that more than 200 kinds of them are now in preclinical or clinical trials [8, 9].

Parthenolide (Figure 2A) as a natural sesquiterpene lactone, is mainly extracted from the herbal plant *Tanacetum parthenium* and has exhibited anti-tumor activities on various tumors including lung, leukemia, pancreatic and breast cancer [10]. Though its effect on lung cancer has ever been reported, its influence on NSCLC and oncogenic drivers of NSCLC was little known. This study was designed to further investigate the cytotoxicity of parthenolide against NSCLC cells and illustrate its potential as B-Raf inhibitor, which is a promising therapeutic strategy for NSCLC. The relative mechanism was also discussed in this study.

## RESULTS

### B-Raf and c-Myc were highly expressed in human NSCLC tissues

**Figure 1:**
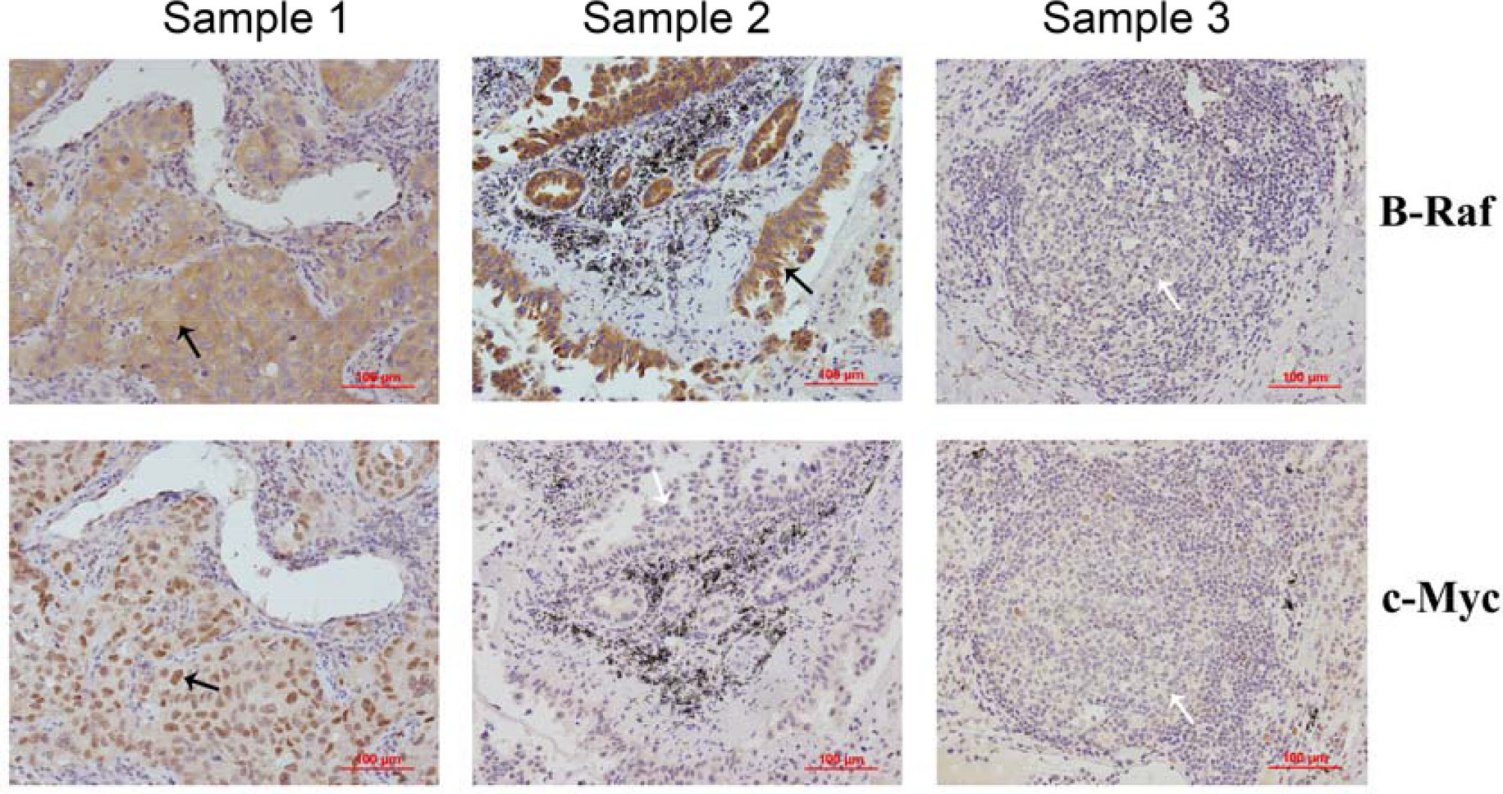
Expression of B-Raf and c-Myc proteins in human NSCLC samples The level of B-Raf and c-Myc in 50 human lung adenocarcinoma samples were examined by immunohistochemistry with specific antibodies. Three representative samples are shown, where either B-Raf or c-Myc staining were negative (white arrow) and positive (black arrow).

Expression of B-Raf and c-Myc (common mutated gene in many cancers) in NSCLC were investigated by immunohistochemistry (IHC) analysis. Figure 1 showed the positive and negative expression of them. B-Raf was highly expressed in 33 out of 50 cases with positive expression rate of 88.0%, which suggested that B-Raf is a promising oncogenic driver for molecular-targeted therapy. High Expression of c-Myc was also found in human NSCLC tissues with positive expression rate of 76.0%. Statistical analysis results based on age, gender, histological grade and stage were summarized in Table 1.

**Table 1:** B-Raf and c-Myc expression with clinicopathological variables in 50 NSCLC samples

Clinicopathological parameters		*n*	B-Raf	*P*	c-Myc	*P*
Age(years)	≥ 60	29	25	0.986^#^	24	0.189
	< 60	21	19		14	
Gender	Male	33	30	0.673^#^	25	1.000^#^
	Female	17	14		13	
Histological Grade	Well-differentiate	3	3	1.000*	1	0.179*
	Moderately-differentiate	27	24		22	
	Poor-differentiate	20	17		15	
Stage	0	1	1	0.244*	0	0.292*
	I	23	22		19	
	II	22	17		16	
	III	4	4		3	

# continuity correction Chi-square

* Fisher's Exact Test.

### Parthenolide and other sesquiterpene lactones showed potent cytotoxicity against human NSCLC cells

**Figure 2:**
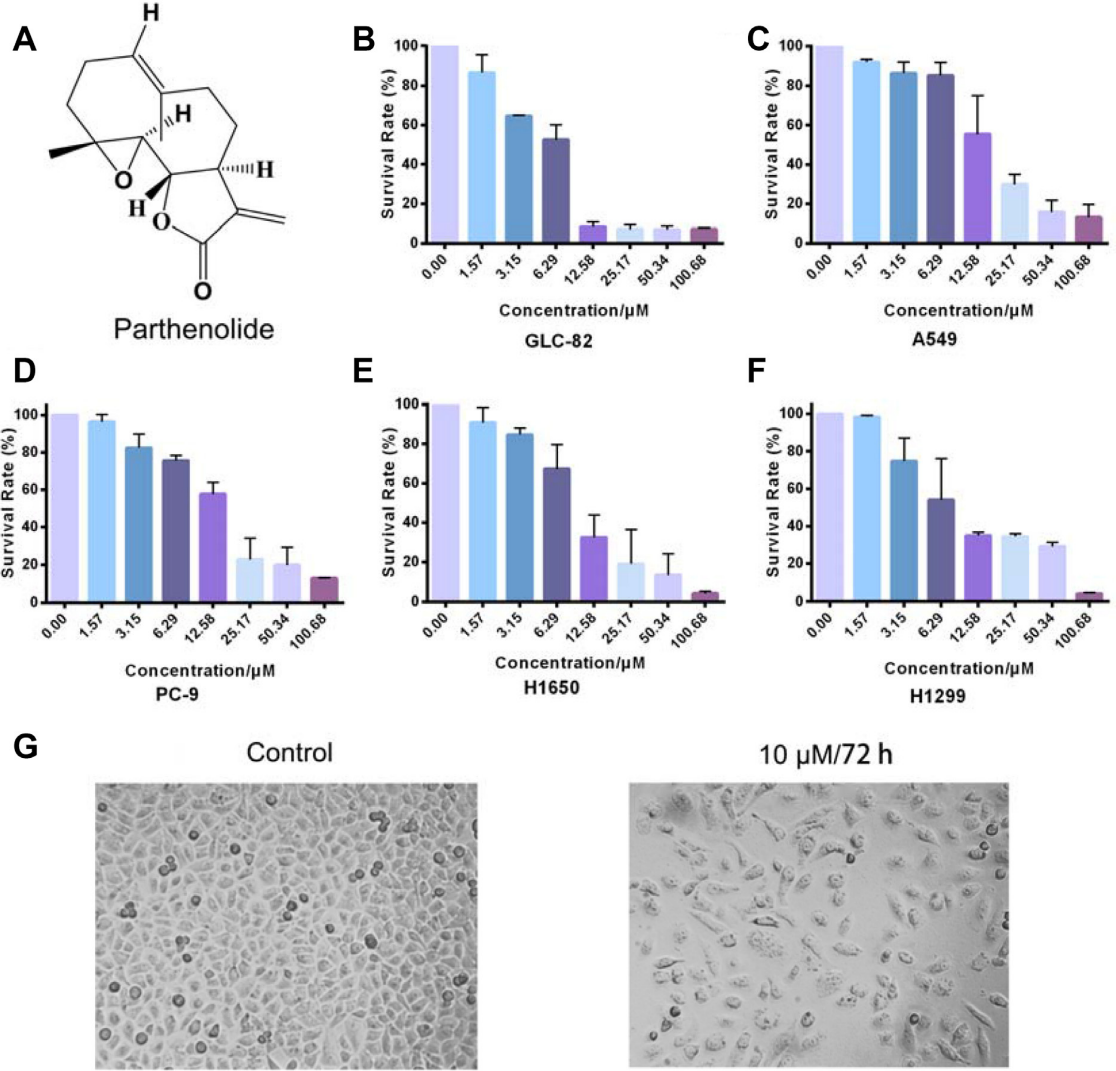
Chemical structure of parthenolide and its effect on different NSCLC cells (**A**) Chemical structure of parthenolide (molecular weight: 248.32 g/mol); (**B**–**F**) Parthenolide inhibited GLC-82, A549, PC-9, H1650 and H1299 cells survival. Survival (%) = (mean experimental absorbance/mean control absorbance) × 100%; (**G**) GLC-82 cell status before and after parthenolide treatment.

MTT assays were carried out with a variety of human lung cancer cells to test the activity of parthenolide and other sesquiterpene lactones. Human lung cancer cells consisted of five NSCLC cell lines, GLC-82, A549, H1650, H1299 and PC-9 cells. As results showed in Figure 2B–2F, parthenolide exhibited potent cytotoxicity towards GLC-82, A549, PC-9, H1650 and H1299 cells, with IC50 values of 6.07 ± 0.45, 15.38 ± 1.13, 15.36 ± 4.35, 9.88 ± 0.09 and 12.37 ± 1.21 μM, respectively. Among them, parthenolide showed the strongest activity against GLC-82 cells. Therefore, GLC-82 cells were chosen for further research. Cell status before and after parthenolide treatment was revealed in Figure 2G. Dabrafenib as positive control and other sesquiterpene lactones were also investigated to elucidate their IC50 values against NSCLC cell lines, which were listed in Table 2. Parthenolide, with the strongest potential, was thus selected for further research with GLC-82 cells.

**Table 2:** Sesquiterpene lactones and Dabrafenib cytotoxicity to NSCLC cell lines

Compound	IC50 (μM)				
	GLC-82	A549	H1650	PC-9	H1299
Dehydrocostus lactone	12.36 ± 1.89	24.40 ± 1.38	30.79 ± 1.20	14.30 ± 1.49	15.34 ± 1.17
Epoxymicheliolide	11.13 ± 0.35	24.51 ± 4.94	24.23 ± 3.84	32.91 ± 2.97	28.44 ± 1.07
Micheliolide	11.32 ± 2.59	22.90 ± 1.33	19.81 ± 0.92	22.82 ± 0.89	19.03 ± 1.81
Arglabin	6.66 ± 0.04	17.09 ± 1.91	21.48 ± 4.14	19.96 ± 0.55	20.22 ± 1.61
Isoalantolactone	17.09 ± 1.68	17.12 ± 1.62	ND	ND	ND
Alantolactone	35.99 ± 5.55	32.89 ± 5.60	ND	ND	ND
Dabrafenib (IC50 nM)	6.12 ± 0.98	5.49 ± 0.77	1.45 ± 0.24	2.19 ± 0.26	1.42 ± 0.33

ND: not detected.

### Parthenolide inhibited migration, proliferation in GLC-82 Cells

**Figure 3:**
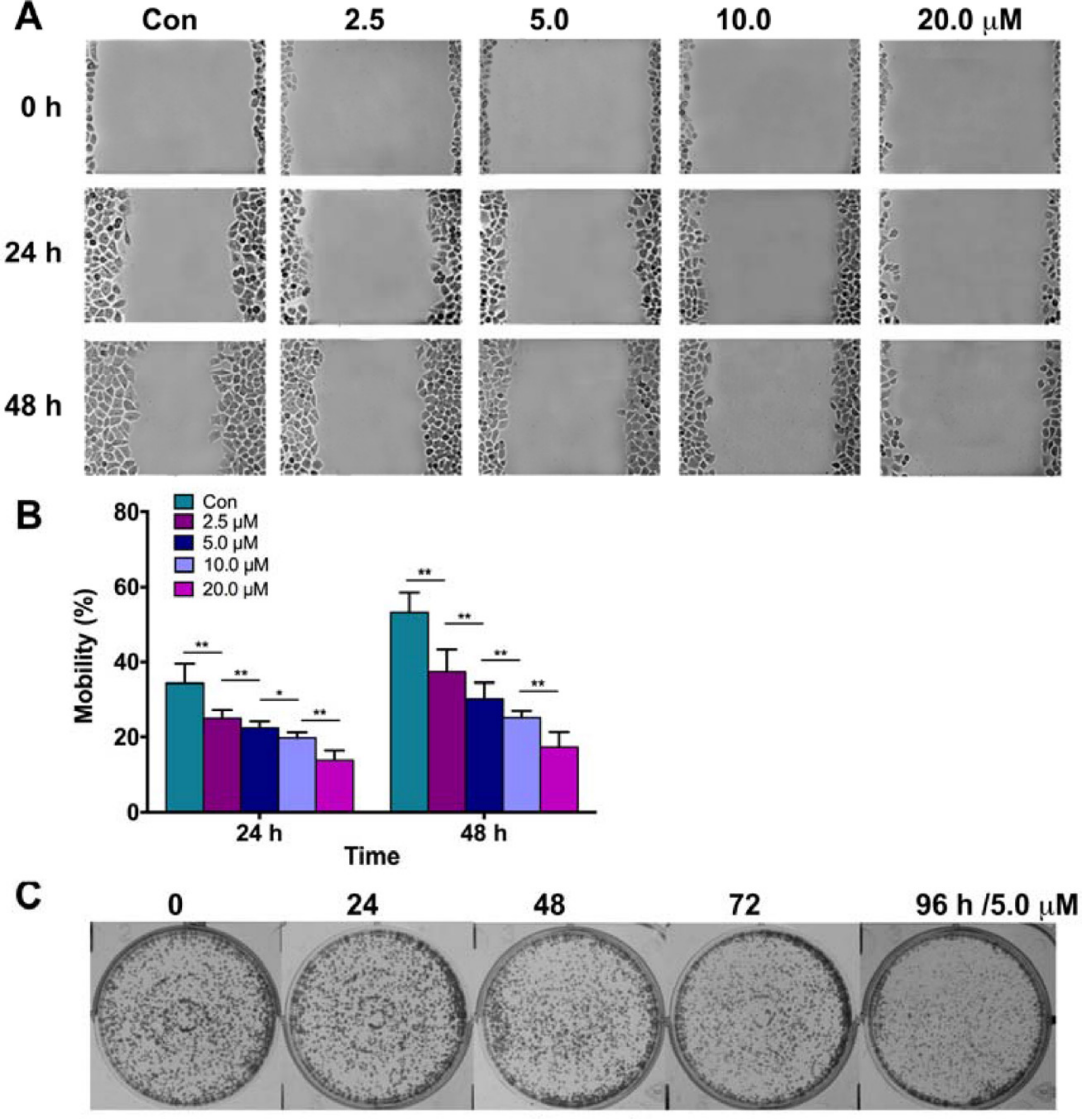
Effects of parthenolide on migration and colon formation of NSCLC cells (**A**) GLC-82 cells migration was inhibited by parthenolide in dose-dependent manner; (**B**) Data analysis of (A). (**C**) Parthenolide reduced colon formation of GLC-82 cells. NS = no significance, *p < 0.05, **p < 0.01, *n* ≥ 3.

As mentioned above, parthenolide exerted potent inhibition on cell growth in different lung cancer cells, especially GLC-82 cells. To further demonstrate its effect on migration and proliferation, scratch wound healing assay and clone formation assay were carried out. Results revealed that parthenolide inhibited wound healing of the cells in time and dose-dependent manners (Figure 3A, 3B) and suppressed clone formation time-dependently (Figure 3C). It's suggested that parthenolide could inhibit human NSCLC cell line GLC-82 migration, proliferation on the basis of its cytotoxicity.

### Parthenolide induced apoptosis in GLC-82 cells in dose-dependent manners

**Figure 4:**
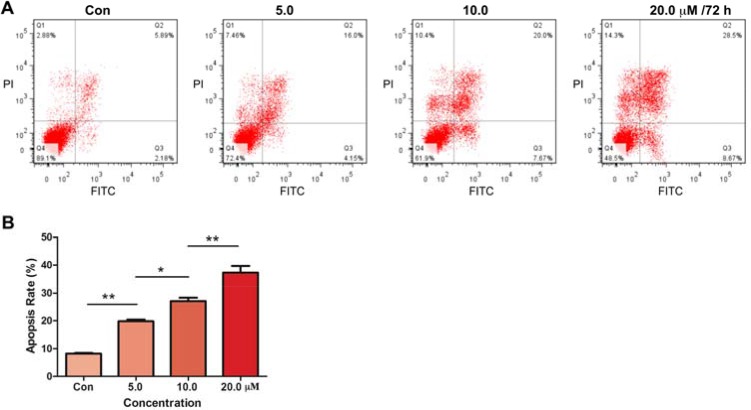
Effects of parthenolide on apoptosis of NSCLC cells (**A**) Parthenolide concentration-dependently induced GLC-82 cells apoptosis, detected by Annexin V-FITC/PI double staining; (**B**) Data analysis of (A). *p < 0.05, **p < 0.01, *n* ≥ 3.

To further confirm whether parthenolide took effects by inducing apoptosis, Annexin V-FITC/PI double staining was carried out. As shown in Figure 4, parthenolide induced GLC-82 cells apoptosis along with the increase of drug concentration. The apoptosis rates of control, 5.0, 10.0 and 20.0 μM parthenolide against GLC-82 cells were 8.21 ± 0.21%, 19.82 ± 0.62%, 27.17 ± 1.20% and 37.30 ± 2.41%, respectively.

### Parthenolide downregulated the expression of B-Raf, c-Myc and phosphorylation of MEK, Erk in GLC-82 cells

**Figure 5:**
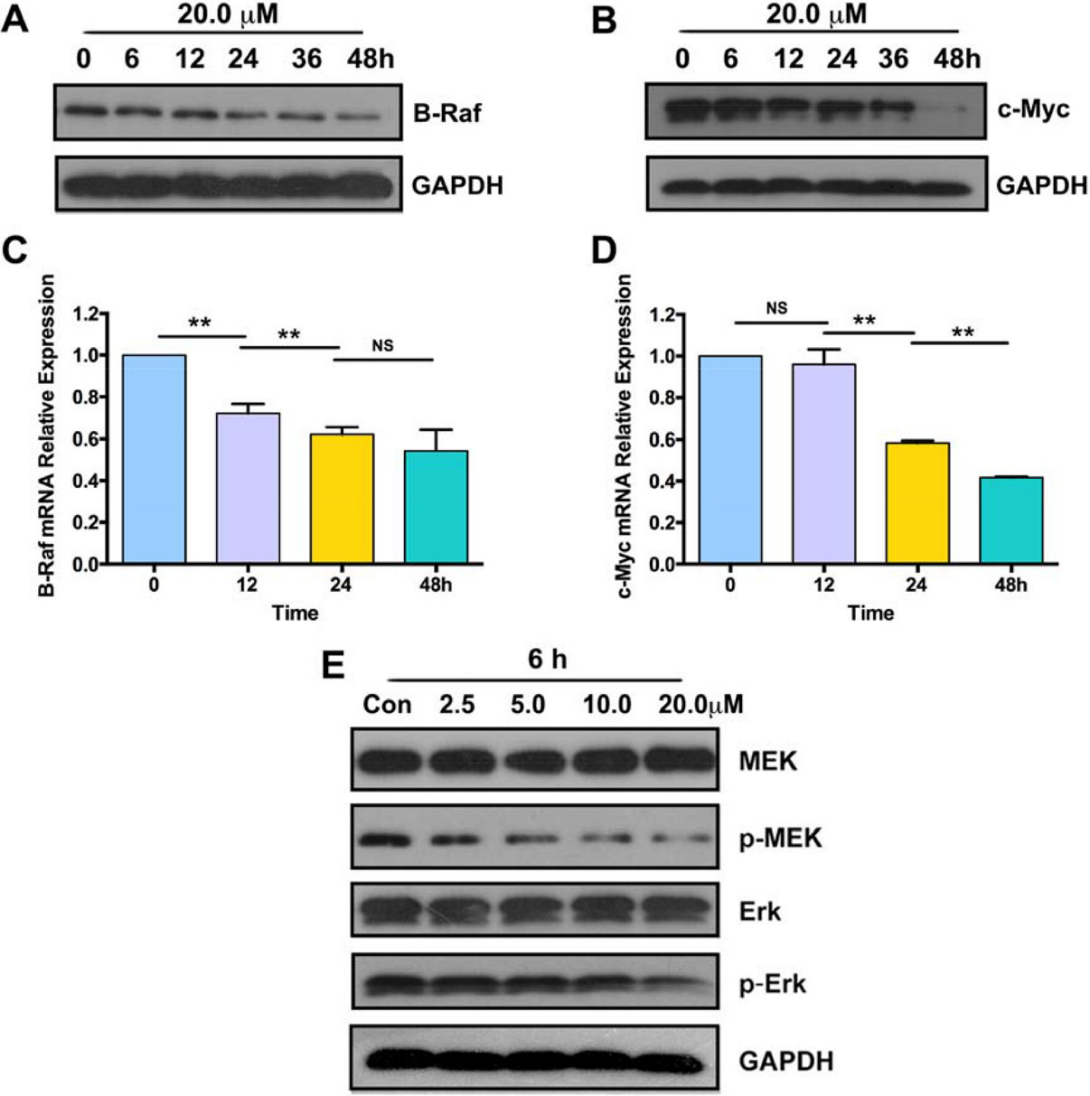
Parthenolide suppressed expression of B-Raf, c-Myc and phosphorylation of MEK, Erk (**A**, **B**) Protein expression of B-Raf and c-Myc after treatment with 20.0 μM parthenolide for 0, 6, 12, 24, 36 and 48 h; (**C**, **D**) B-Raf and c-Myc mRNA levels detected by RT-QPCR after exposure of 20.0 μM parthenolide for indicated time; (**E**) Level of MEK, p-MEK, Erk, p-Erk detected after treatment with 0, 2.5, 5.0, 10.0, 20.0 μM parthenolide for 6 h. NS = no significance, *p < 0.05, **p < 0.01, *n* ≥ 3.

To investigate the potential of parthenolide as B-Raf inhibitor, western blot and RT-QPCR were applied for detection. When GLC-82 cells were treated with 20.0 μM parthenolide for 0-48h, the expression of B-Raf in protein (Figure 5A) and mRNA (Figure 5C) level decreased in turn. Expression of c-Myc was also measured in the same way. When GLC-82 cells were treated with the longer medication time, protein and mRNA level of c-Myc were lower (Figure 5B, 5D). What's more, phosphorylation of MEK and Erk was suppressed after exposure to different concentration of parthenolide for 6 h, while the total protein level of MEK and Erk didn't change (Figure 5E).

### Parthenolide suppressed MAPK/Erk pathway signaling in GLC-82 Cells

**Figure 6:**
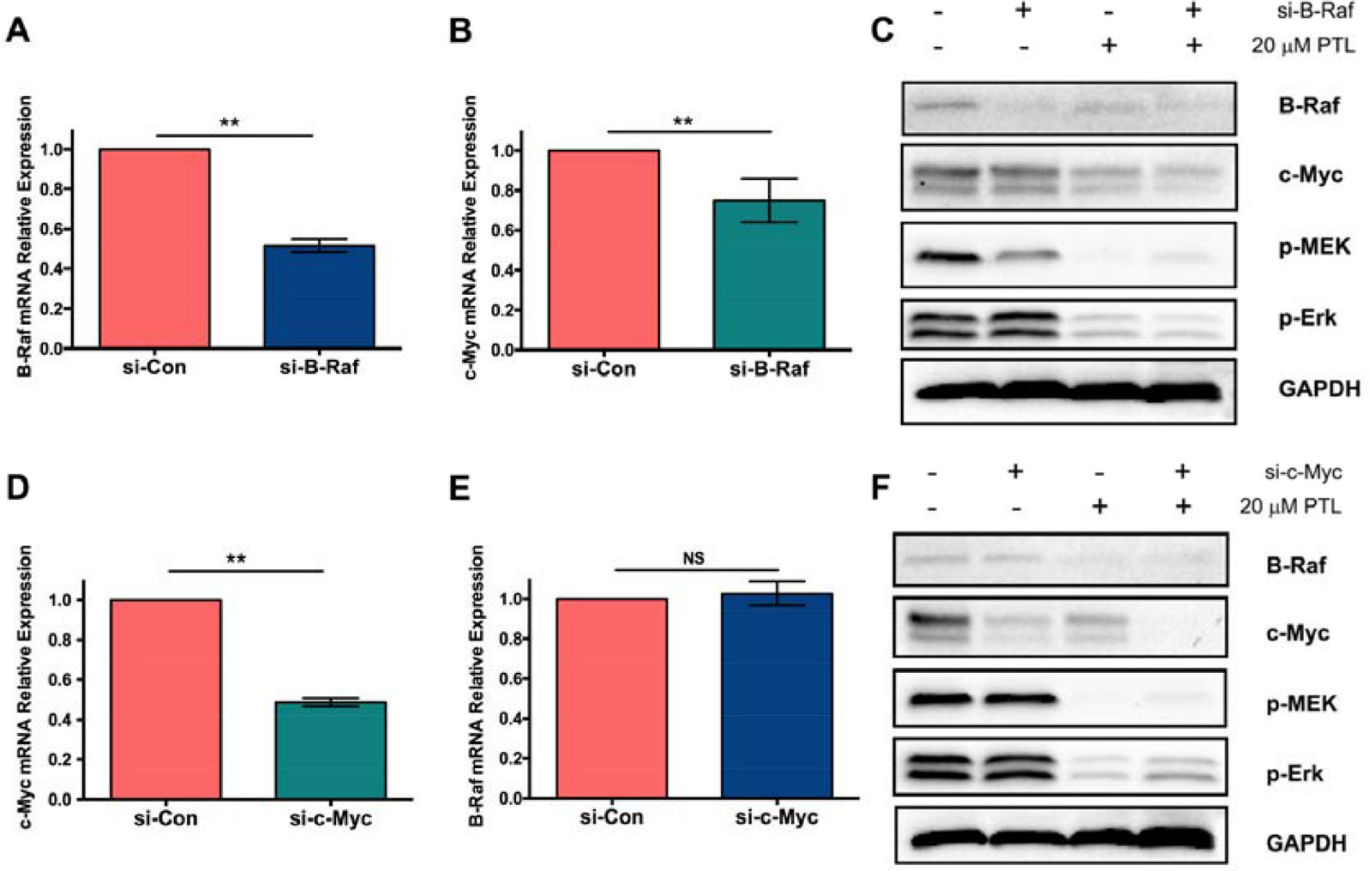
Effect of parthenolide on MAPK/Erk pathway signaling and c-Myc expression (**A**) Interference confirmed by RT-QPCR after si-B-Raf transfection; (**B**) c-Myc mRNA expression after transfection with si-B-Raf; (**C**) Level of different proteins detected by western blot after treatment with si-B-Raf and/or parthenolide; (**D**) Interference confirmed by RT-QPCR after si-c-Myc transfection; (**E**) B-Raf mRNA expression after transfection with si-c-Myc; (**F**) Level of different proteins detected by western blot after treatment with si-c-Myc and/or parthenolide. NS = no significance, *p < 0.05, **p < 0.01, *n* ≥ 3.

It was found above that parthenolide could inhibit the expression of B-Raf and c-Myc. To further study the interaction between them and their effect on MAPK/Erk pathway, siRNA-interference technique was employed. The results exhibited that the expression of c-Myc, p-MEK and p-Erk were downregulated after the transfection of B-Raf siRNA and further downregulation was found when combined with parthenolide (Figure 6A, 6C). On the other hand, levels of B-Raf, p-MEK and p-Erk showed no changes after the transfection of c-Myc siRNA (Figure 6D, 6F). They were further confirmed in mRNA level by RT-QPCR, as shown in Figure 6B, 6E. It's speculated that parthenolide targeted on B-Raf and inhibited the MAPK/Erk pathway signaling. C-Myc was probably the further downstream of MAPK/Erk pathway, which had no feedback on the B-Raf expression and MAPK/Erk signaling.

### Parthenolide decreased STAT3 activity and had no changes in GSK3α/β and Akt

**Figure 7:**
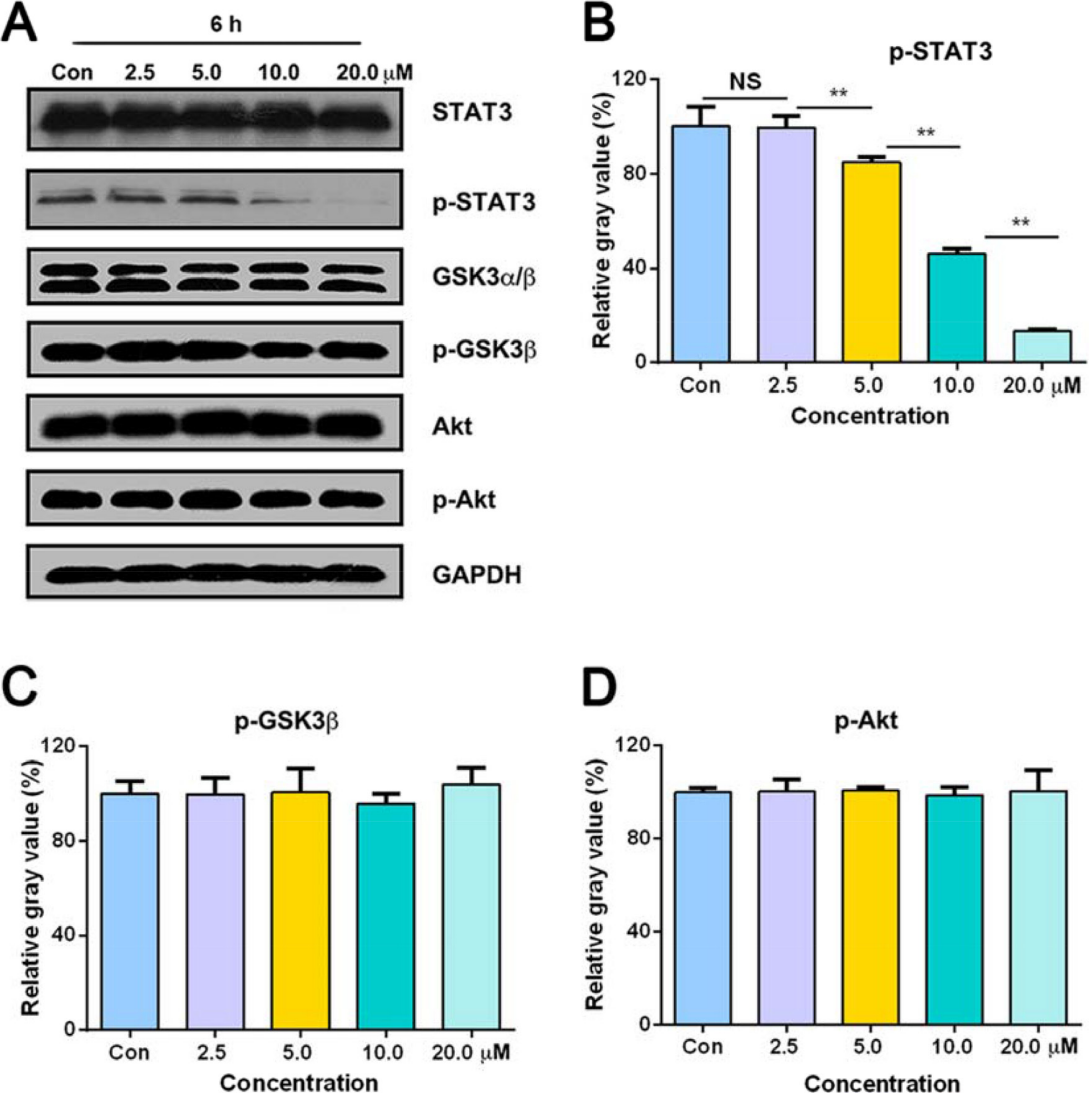
Total and phosphorylated levels of STAT3, GSK3α/β and Akt after treatment with 0, 2.5, 5.0, 10.0, 20.0 μM parthenolide for 6 h (**A**) Expression of STAT3, p-STAT3, GSK3α/β, p-GSK3β, Akt and p-Akt in GLC-82 cells. (**B**, **C**, **D**) Gray intensity analysis of Western blot results of p-STAT3, p-GSK3β and p-Akt. NS = no significance, *p < 0.05, **p < 0.01, *n* ≥ 3.

Other than the above findings, parthenolide was also found to inhibit the phosphorylation of STAT3, which may partly attribute to the potent activity of parthenolide. After treatment with 2.5, 5.0, 10.0, 20.0 μM parthenolide, p-STAT3 level expressed as gray value was 99.47 ± 4.90, 84.98 ± 2.16, 45.86 ± 2.18, 13.40 ± 0.71% of control, respectively (Figure 7A, 7B). On the other hand, parthenolide had no influence on both total and phosphorylated level of Akt and GSK3α/β. After exposure to parthenolide of 2.5, 5.0, 10.0, 20.0 μM parthenolide, p-GSK3β level was 99.79 ± 6.86, 100.58 ± 10.05, 95.80 ± 4.05, 103.79 ± 7.17 % of control, respectively (Figure 7A, 7C). After GLC-82 cells were treated with 2.5, 5.0, 10.0, 20.0 μM parthenolide, p-AKT level was 100.32 ± 5.35, 100.88 ± 1.22, 98.74 ± 3.68, 100.61 ± 8.83% of control, respectively (Figure 7A, 7D).

## DISCUSSION

B-Raf, short for v-Raf murine sarcoma viral oncogene homolog B, is a member of serine/threonine-specific protein kinases [11, 12]. Different types and frequency of B-Raf mutations associated with human cancers have been identified [13–16]. Notably, B-Raf was also considered as one of the oncogenic drivers for NSCLC with frequency of about 3% [17–19]. Differentially high expression of B-Raf in human NSCLC tissues was confirmed by immunohistochemistry in our study too, with positive expression rate of 88.0% (Figure 1). The rate in our IHC analysis was higher than that of the published records and this is probably because the NSCLC tissues we examined are all lung adenocarcinoma tissues which were reported to have a higher frequency of B-Raf mutation. Our results showed that B-raf or c-Myc positive rates exhibited no difference when age, gender, histological grade and clinical stage were concerned. It's unknown whether the difference will be significant when the number of clinical samples is more than 50.

Drugs that treat cancer driven by B-Raf mutations have been developed, such as Vemurafenib and Dabrafenib. These two drugs are approved by FDA for treatment of late-stage melanoma and B-Raf-mutant lung adenocarcinomas. It's proved that B-Raf inhibitors improved rates of overall and progression-free survival in patients and showed potent potential in treatment of B-Raf-mutant cancers [20–22]. In this term, B-Raf inhibitors were the potential strategy for NSCLC treatment. The active compound of feverfew (*Tanacetum parthenium*), parthenolide, has shown potential significant cancer suppression activity *in vitro* and *in vivo*. Although the effect of parthenolide as an inhibitor of NF-κB activity has been reported previously in several cancers, its significance, detailed mechanism as B-Raf inhibitor for NSCLC has not yet been investigated [23, 24].

MTT assays, in this way, were firstly carried out to test the cytotoxicity of parthenolide to different human NSCLC cells. In consistence to its effect on other cancer types, parthenolide also exhibited potent inhibition to NSCLC cells, especially GLC-82 cells (Figure 2). Furthermore, parthenolide could inhibit human NSCLC cell line GLC-82 migration, proliferation and induce apoptosis on the basis of its cytotoxicity (Figures 3, 4).

Members of the Raf family encode serine/threonine protein kinases and play a role in regulating the MAP kinase (MAPK)/ ERKs signaling pathway through direct interaction and phosphorylation. The canonical MAPK/Erk pathway is composed of three types of MAPKKK: A-Raf, B-Raf and C-Raf kinases. B-Raf is the gene most commonly mutated at this level in human cancer and shown to display higher MEK kinase activity than other members [25]. Western blot and RT-QPCR results showed that parthenolide suppressed expression of B-Raf in both protein and mRNA levels (Figure 5A, 5C). Combined with the IHC results (Figure 1), it's preliminarily proposed that parthenolide is able to target on B-Raf mutation and then inhibit the development of NSCLC including proliferation and invasion.

Levels below B-Raf of the MAPK cascade are MAPKKs, which are composed of MEK1 and MEK2. Erk1 and Erk2 are the further downstream and the final effectors of the MAPK pathway [26]. As shown in Figure 5E, phosphorylation of MEK and Erk was decreased after exposure to different concentration of parthenolide for 6 h, while the total protein level of MEK and Erk didn't change. Erk phosphorylation can lead to activation of multiple substrates that are responsible for stimulation of cell proliferation. Spatial localization of ERK determines target substrates and later effects within the cell [27]. When located at nucleus, active Erk causes phosphorylation and activation of various transcription factors such as c-Fos, c-Jun, Elk-1, c-Myc and ATF2 promoting cell progression [28]. For one thing, as GLC-82 cells were treated with the longer medication time, protein and mRNA level of c-Myc were lower in our study (Figure 5B, 5D). For another thing, parthenolide had no influence on both total and phosphorylated level of GSK3α/β (Figure 7). These suggested that parthenolide might inhibit Erk to locate at nucleus and thereby downregulate the expression of c-Myc in human NSCLC GLC-82 cells.

To illustrate whether parthenolide specifically target on B-Raf and the relationship between B-Raf and c-Myc, siRNA interference was taken. Results (Figure 6) indicated that parthenolide specifically targeted on B-Raf and then inhibited the MAPK/Erk pathway signaling. c-Myc, on the other hand, was probably the further downstream of MAPK/Erk pathway, which had no feedback on the B-Raf expression and MAPK/Erk signaling. However, further research about the interaction between them are still in need and will be taken next.

STAT proteins are extracellular ligand-responsive transcription factors that mediate broadly diverse biological processes, including cell proliferation, transformation, apoptosis and differentiation. STAT protein, especially STAT3, are usually phosphorylated aberrantly in tumors, inducing malignant proliferation and apoptosis inhibition [29]. It's reported that parthenolide can inhibit STAT3 activity and therefore suppress the development of tumor [30]. Parthenolide, in our study, was found to inhibit the phosphorylation of STAT3 (Figure 7), which probably attribute to the potent activity of parthenolide. Deregulation of PI3K/protein kinase B (PKB/AKT)/mammalian target of rapamycin (mTOR) (PI3K) pathway and MAPK pathway frequently occurs in human cancers. Several studies demonstrated that blockade of one pathway may lead to the activation of the other signaling cascade [31]. Akt, as the critical factor of PI3K pathway, was examined in our research too. Interestingly, parthenolide had no influence on both total and phosphorylated level of Akt, which indicated that the effect of parthenolide on MAPK pathway didn't activate the PI3K pathway signaling. The potent cytotoxicity of parthenolide may be also explained in this way.

## MATERIALS AND METHODS

### General experimental procedures

Parthenolide (PTL), Dehydrocostus, Epoxymicheliolide, Micheliolide, Arglabin, Isoalantolactone and Alantolactone with purity of HPLC ≥ 98% were purchased from Nanjing Spring & Autumn Biological Engineering Co., Ltd (Nanjing, China). Dabrafenib was purchased from MCE Co.,Ltd.(Monmouth Junction, NJ). RPMI 1640, DMEM and Opti-MEM (1×) medium (Gibco, Carlsbad, CA), Block it^™^ Alexa Fluor^®^ Red Fluorescence Control and Lipofectamine^®^ 2000 (Invitrogen, Carlsbad, CA), TRIzol^®^ Reagent (Ambion, Carlsbad, CA) were purchased from Thermo Fisher Scientific Inc.. Fetal bovine serum (FBS) were bought from Zhejiang Tianhang Biotechnology Co.,Ltd. (Hangzhou, China). 3-(4,5-dimethylthiazolyl-2)-2,5-diphenyl tetrazolium bromide (MTT) was obtained from MP Biomedicals Inc.. PrimeScript^™^ RT Master Mix and SYBR^®^ Premix Ex Taq^™^ (Tli RNaseH Plus) were purchased from Takara Bio Inc. (Dalian, China). The following antibodies were used: anti-Erk 1/2, anti-p-Erk 1/2, anti-MEK, anti-p-MEK, anti-GSK3α/β, anti-GSK3β, anti-c-Myc, anti-Akt, anti-Akt (Ser 473), anti-STAT3, anti-p-STAT3 (Cell Signaling Technology, Inc.), anti-B-Raf (Abcam Inc., Cambridge, UK), anti-GAPDH (Bioworld Technology Inc., St Louis Park, MN). Primers for B-Raf, c-Myc and GAPDH were synthesized by Invitrogen Inc.(Carlsbad, CA).

### Cell culture

GLC-82, A549 cell lines were kindly provided by Prof. Li-wu Fu (Sun Yat-sen University, Guangzhou, China); H1650, H1299, PC-9 cells were presented by Prof. Zhi Shi (Jinan University, Guangzhou, China). GLC-82, A549 cells were cultured in RMPI 1640 medium and H1650, H1299, PC-9 cells were maintained in DMEM medium at 37°C in a 5% CO_2_ incubator. Both mediums were supplemented with 10% FBS and 100 U/mL each of penicillin and streptomycin [32].

### Human tissue preparation

Human lung tumor tissues were collected from 50 patients between 2011 and 2014 at Shanxi Provincial People's Hospital. The patients (males, *n* = 34; females, *n* = 16) ranged from 42 to 81 years of age and accepted no chemotherapy before operation. The tissue samples were immediately fixed in 4% paraformaldehyde in PBS and was embedded in paraffin for immunohistochemistry. All participants provided written informed consent. The study conformed to the ethical guidelines and was approved by the hospital Ethics Committee [33].

### MTT assay

The effects of parthenolide on different NSCLC cells viability were estimated using MTT. All the five cell lines were respectively seeded in 96-well plates for 24 h and treated with parthenolide with gradient concentration for 72 h. MTT solution was then added for 4h. Absorbance at 540/655 nm was recorded using Epoch Microplate Spectrophotometer (BioTek Instruments, Inc.). Cell survival was calculated with the following formula: survival (%) = (mean experimental absorbance/mean control absorbance) × 100% [34].

### Scratch wound healing assay

For scratch experiment, GLC-82 cells were seeded on six-well plates and cells were allowed to reach confluence overnight. Subsequently, a 200 μL pipette tip was used to scratch the cell monolayer and the wounded cell layer was washed to remove loose cells. Medium containing graded of parthenolide were added to the plates. After 0, 24 and 48 h culture, images were captured. Cell motility was determined according to the percentage of the repaired area [35].

### Clone formation assay

GLC-82 cells were seeded into six-well plates at a density of 2000 cells per well. After treatment with parthenolide for indicated time, cells were washed with PBS, fixed in methanol for 15 min, and stained with 0.5% crystal violet for 15 min. Visualized colonies were then photographed [36].

### Annexin V-FITC/PI apoptosis detection

Apoptosis rates were quantified by Annexin V-FITC/PI apoptosis detection kit (KeyGEN, Nanjing, China) according to the manufacturer's protocol. Briefly, after cells were treated with indicated concentrations of parthenolide for 72 h, cells were collected and washed twice with ice-cold PBS. Then 5 × 10^5^ cells were resuspended in 0.5 mL binding buffer containing Annexin-V (1:50) and 40 ng/sample of PI for 30 min at 37°C in the dark. Subsequently, the cells were determined by flow cytometer (Becton Dickinson, NY) and analyzed by CellQuest software. At least 10,000 cells were analyzed for each sample. The apoptosis rate (%) = (the number of apoptotic cells/the number of total cells observed) × 100% [37].

### Western blotting analysis

Cultured cells were collected in lysis buffer (Cell Signalling Technology, Danvers, MA). Equal amounts of proteins were separated on 10–12% sodium dodecyl sulfatepolyacrylamide gel electrophoresis (SDS-PAGE) and transferred onto PVDF membranes (Millipore, Boston, MA). Membranes were blocked with 5% non-fat milk, incubated with primary antibodies at 4°C overnight. After incubation with horseradish peroxidase (HRP) conjugated secondary antibodies for 2 h, blots were revealed by enhanced chemiluminescence procedures according to the manufacturer's protocol [38].

### RNA interference and infection

SiRNA sequences targeting B-Raf, c-Myc and a non-targeting control were purchased from Cell Signaling Technology, Inc. and Santa Cruz Biotechnology, Inc. (Santa Cruz, CA). Transfection was performed according to the manufacturer's instructions. GLC-82 cells were seeded in six-well plates and transfected transiently with siRNA using Lipofectamine^®^ 2000 (Invitrogen, Carlsbad, CA). After transfection for 24 h, GLC-82 cells were used for Western blot and RT-QPCR [39].

### Real-time quantitative reverse transcription-PCR

Total RNA was extracted from cells using TRIzol^®^ Reagent (Ambio, CA), and 500 ng RNA per 10 ul reaction solution was used to synthesize cDNA using the PrimeScript^™^ RT Master Mix (Takara, Japan). cDNA was amplified and quantified by RT-QPCR with SYBR^®^ Premix Ex Taq^™^ (Tli RNaseH Plus) (Takara, Japan). GAPDH was used as the internal control. The primer sequences for the PCR amplification of B-Raf, c-Myc and GAPDH were: B-Raf primer set (forward, 5′-AGA AAG CAC TGA TGA TGA GAG G-3′; reverse, 5′-GGA AAT ATC AGT GTC CCA ACC A-3′), c-Myc primer set (forward, 5′-GCT CAT TTC TGA AGA GGA CTT GT-3′; reverse, 5′-AGG CAG TTT ACA TTA TGG CTA AAT C-3′), and GAPDH primer set (forward, 5′-GGA AGG TGA AGG TCG GAG TCA-3′; reverse, 5′-GTC ATT GAT GGC AAC AAT ATC CAC T-3′) [40].

### Immunohistochemistry

Paraffin-embedded tumor tissues were sectioned and deparaffinized with xylene. The slides were immersed into different concentrations of alcohol for rehydration and then in 3% H_2_O_2_ to block endogenous peroxidase. After washing with distilled water, the slides were incubated with different grades of antibody. Immunoreactions were visualized using DAB substrate, counterstained by hematoxylin and cover-slipped for microscopic examination [41].

### Statistical analysis

Each experiment was repeated at least three times. All numerical data were presented as mean ± standard deviation. Statistical difference in each assay was analyzed by Graphpad Prism 6, and was tested for significance using *t* test and ANOVA analysis of variance. *P* < 0.05 was considered significant.

## CONCLUSIONS

**Figure 8:**
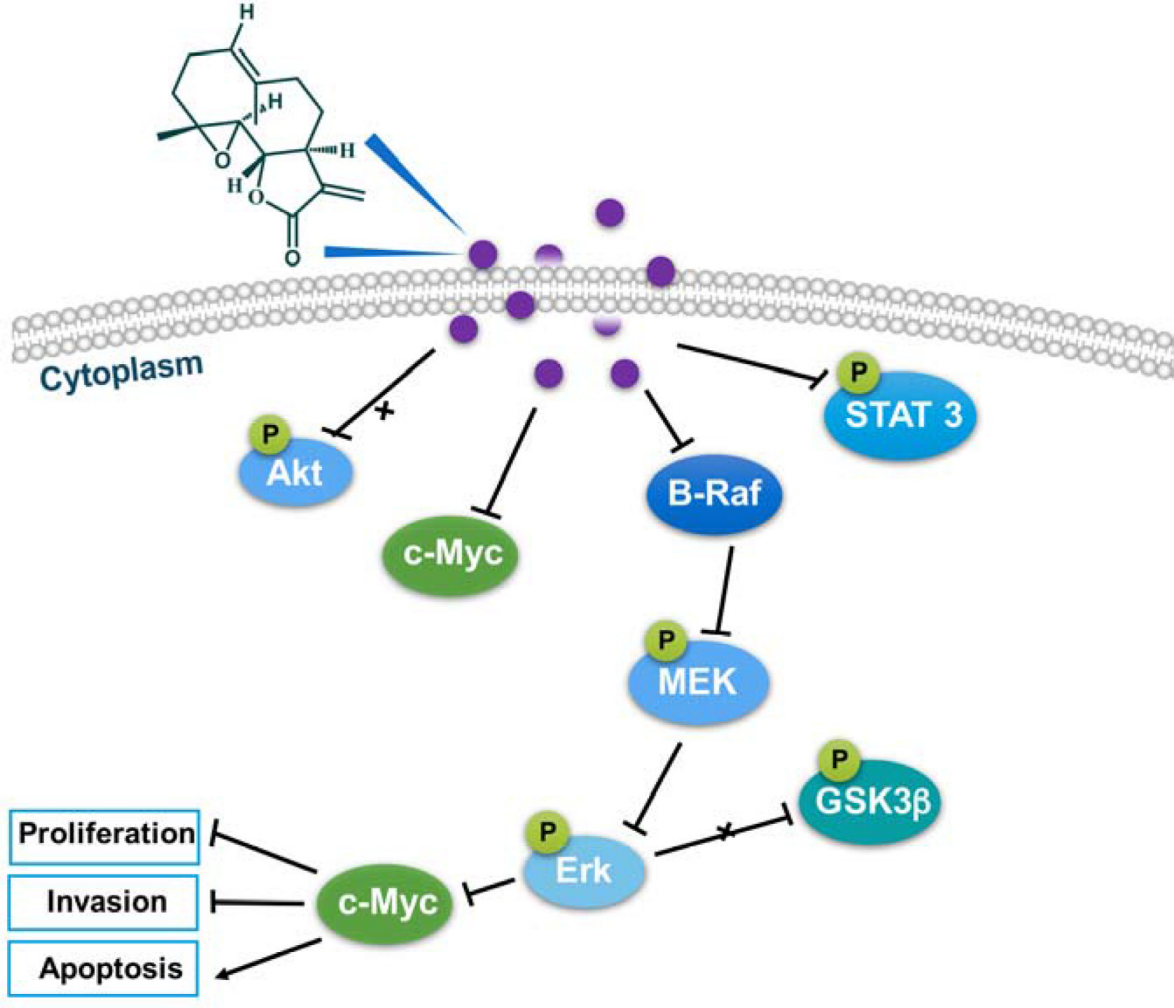
The involved mechanism of parthenolide against NSCLC cells

In conclusion, parthenolide exhibited potent cytotoxicity towards human NSCLC cells. Parthenolide could target on B-Raf and inhibited the MAPK/Erk pathway signaling which is independent of PI3K pathway. Inhibition of STAT3 phosphorylation was also found to be related with the potent cytotoxicity of parthenolide. The involved mechanism was summarized in Figure 8.
